# Selected

**Published:** 1909-02

**Authors:** 


### Selected.

### The Yearly Toll.

A review of this year's football season indicates about the same results as regards the killed and wounded on the field of battle, showing that our institutions of learning are still maintaining their usual high standard of moral and literary excellence. This is due to the fact that the faculties of the various colleges are realizing the importance of practical training and more utilitarian methods. By ignoring the three R's, mathematics, literature and the like, and exacting less frequent attendance at recitations the candidate for the degree of A. B. is enabled to devote a good part of his time to athletics and team work and thus fit himself for the duties and responsibilities of his future career. Many a minister owes his success to the skillful use of the low tackle. The lawyer who can politely and accurately butt the opposing counsel in the stomach is surely to enjoy a certain prestige, while the physician who appreciates the advantages of the strangle hold can acquire in this way a much better control of his patient. Inasmuch as physical training is so markedly in the ascendant, it is no more than just that the physical instructor, the coach and the strong man should enjoy full professorships and that the chairs which are purely scholastic should be abolished.

While various bodily ills of greater or less magnitude are attendant upon a proper and up-to-date collegiate training, they should be ignored, for are they not the penalties of greatness? What is invalidism or disability to one who has made a record, and how trivial seems a hypertrophied heart to the winner of the Olympic games?—*New England Medical Gazette.*

That is what Josh Billings calls *"Sarkassum."*—ED.

### Texas Women and Tuberculosis.

The Federated Women's Clubs of Texas, at its recent meeting at San Angelo, passed resolutions for the establishment of a State tuberculosis sanitarium. The resolutions were addressed to the Legislalure, and charged that since medical science had declared tuberculosis the leading sanitarium problem of the day, since climatic conditions of Southwest Texas were a drawing card for consumptives, then "Be it resolved that the president of the Federation be empowered to appoint a committee to promulgate educational movement on tuberculosis problems, with the enlistment of support of all women's organizations, and that the Federation lend every effort to legislative measures proposed for the relief of tuberculosis sufferers as well as protection of the people."—*Ex.*

### The Independent Press.

From the days of pagan antiquity to the present hour, there has never been a time or country wherein mankind could claim immunity from all persecutions for intellectual differences. But it is outrageous in this day and age of the world that honest, experienced physicians should be vilified, remedies which have proved efficacious for years in the practice of thousands and thousands of our profession accused of being fraudulent and valueless, and independent medical journals censured because they dare to question the authority and fallibility of the journal of the American Medical Association and a few parrot-like State journals, who can drink only out of gourds inscribed with the J. A. M. A. trade-mark.

Never before in the history of medicine has there been such a need of strong, able, fearless, independent men in medical journalism as there is right now.

Stub pencils and paste-pots and blind obedience to certain cliques will suffice no more. Editors with only these qualifications and who have reached the sanctum through the composing room instead of the library, or through the influence of medical politics, are totally unable to subserve the best interests of the physicians of America.

It is especially desirable that a strong, but not offensive individuality should be cultivated in the editorial columns, that the emphasis given to private, yet enlightened, judgment may exert public influence. Upon an editor of a medical journal today it is of all things incumbent that a candid, fair and intelligent discussion of every topic of interest to the profession or to the health of the community should form the ruling motive of effort.

Above all, let us support and encourage the independent medical journal. The official society organs are necessary and have their proper place. But under no circumstances should they be allowed to supplant independent journals.—*Geo. F. Butler, M. D., in Transactions Amer. Med. Ed. Assn.*

A high temperature just after or during an abortion is evidence of intrauterine manipulation, especially if the discharge from the uterus is fetid.—*American Journal of Surgery.*

